# GFRAL is required to mediate changes in systemic metabolism in response to mitochondrial stress in brown adipose tissue

**DOI:** 10.1007/s00109-026-02671-z

**Published:** 2026-04-14

**Authors:** Ayushi Sood, Joshua Peterson, Jayashree Jena, Vamsi Challa, Caden Washburn, Randy J. Seeley, Renata O. Pereira

**Affiliations:** 1 Department of Internal Medicine, University of Iowa, 169 Newton Road, 4322 PBDB, Iowa, IA 52242 USA; 2 Department of Internal Medicine, University of Michigan, Ann Arbor, MI USA

**Keywords:** OPA1, GFRAL, GDF15, Brown adipose tissue, Obesity, Thermoregulation

## Abstract

**Abstract:**

Growth differentiation factor 15 (GDF15) is a cytokine induced in several tissues in response to stress. GDF15 suppresses food intake and increases energy expenditure via its actions on the glial-derived neurotrophic factor receptor α family-like specific receptor (GFRAL), located in the hindbrain. We recently showed that selective deletion of the mitochondrial fusion protein optic atrophy 1 (OPA1) in brown adipocytes (OPA1 BKO) leads to GDF15 secretion, partially mediating resistance to diet-induced obesity (DIO), and improving thermoregulation. To investigate whether GDF15 signaling through GFRAL is necessary to mediate these metabolic effects, we crossed OPA1 BKO mice with GFRAL global knockout mice (DKO). Under isocaloric conditions, DKO mice had similar body weight as control and OPA1 BKO mice. Upon high-fat diet feeding, DKO mice were partially resistant to DIO, but lacked the improvement in glucose homeostasis and insulin sensitivity observed in OPA1 BKO mice. Finally, DKO mice were susceptible to cold-induced hypothermia, suggesting a role for GFRAL in core body temperature regulation in the OPA1 BKO mice. Our data reveals a novel BAT-GDF15-GFRAL axis that modulates resistance to DIO and improves thermoregulation in mice in the context of mitochondrial stress.

**Key messages:**

- OPA1 deletion induces a BAT-GDF15-GFRAL axis to regulate systemic metabolic homeostasis.
- GDF15-signaling through GFRAL partially mediates resistance to DIO in mice lacking OPA1 in BAT.
- GFRAL mediates GDF15’s effects on energy homeostasis in DIO OPA1 BKO mice.
- GDF15-GFRAL signaling is required to maintain core body temperature in cold-exposed OPA1 BKO mice.

**Graphical abstract:**

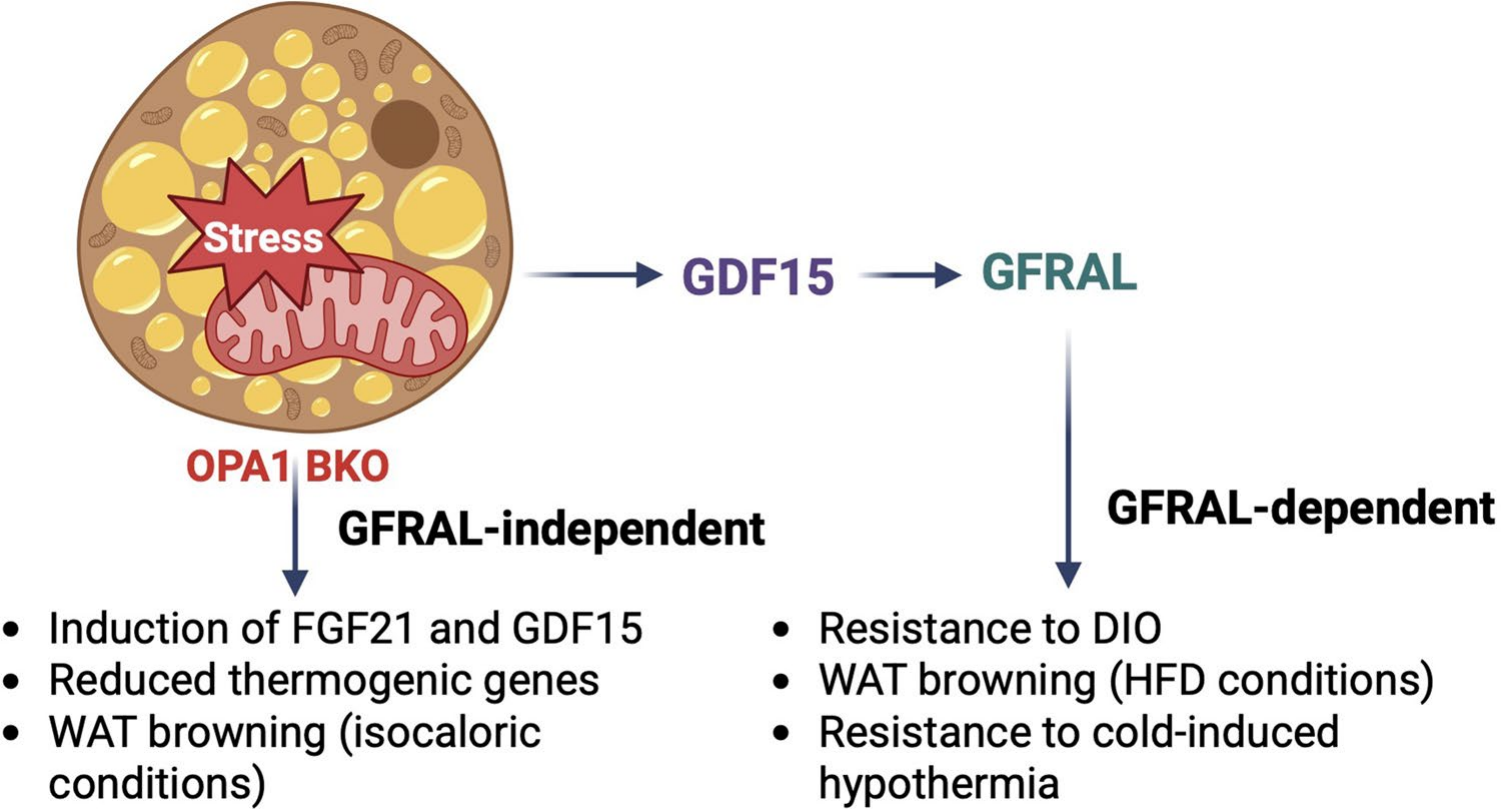

**Supplementary Information:**

The online version contains supplementary material available at 10.1007/s00109-026-02671-z.

## Introduction

The rate of obesity and its associated comorbidities such as type 2 diabetes and cardiovascular disease continue to increase, representing a major public health issue in the United States and globally [1]. The discovery of brown adipose tissue (BAT) in adult humans sparked interest in leveraging BAT thermogenic activity to increase energy expenditure thereby attenuating obesity. In addition to mediating thermogenesis, BAT can secrete signaling molecules, referred to as batokines, that may act in autocrine, paracrine, and endocrine manners to improve systemic metabolic health. Importantly, recent studies in human subjects linked increased BAT activity with reduced prevalence of cardiometabolic diseases and healthier metabolic phenotypes in obesity [2, 3].

Our recent studies showed that in mice lacking the mitochondrial fusion protein optic atrophy 1 (OPA1) in BAT (OPA1 BKO), the activating transcription factor 4 (ATF4) is activated, leading to induction of fibroblast growth factor 21 (FGF21) and growth factor 15 (GDF15) as batokines [4–6]. In this model, while FGF21 was primarily required to mediate induction of compensatory browning of white adipose tissue (WAT), increasing resting metabolic rates and promoting leanness in mice fed isocaloric diet, GDF15 contributed to the resistance to diet-induced obesity (DIO) by driving increases in energy expenditure in OPA1 BKO mice. Furthermore, both, BAT-derived FGF21 and GDF15 were required to maintain core body temperature, with their absence rendering OPA1 BKO mice cold intolerant [5].

GDF15 is a divergent member of the TGF-β superfamily that can be expressed by most cell types in the body in response to stress [2, 3, 7]. Accordingly, plasma concentrations of GDF15 are increased under conditions of physiological stress, such as aging [8] and pregnancy [9], as well as in various disease states such as cancer [3], obesity [10], and cardiovascular disease [11, 12]. GDNF family receptor α-like (GFRAL) was recently identified as the receptor mediating GDF15’s effects on reducing food intake and increasing energy expenditure by activation of the GDF15–GFRAL-RET complex in neurons in strict areas of the hindbrain [13–16]. Intriguingly, although GFRAL is the only well-validated receptor for GDF15, additional receptors have been proposed through which GDF15 may mediate signaling in peripheral tissues in a GFRAL-independent manner [17–21].

To investigate the effects of signaling through GFRAL to mediate the metabolic improvements in OPA1 BKO mice, we crossed GFRAL global knockout mice with OPA1 BKO mice (DKO) and subjected them to 12 weeks of high-fat feeding or acute cold exposure. Our data revealed that, GFRAL is partially required for the resistance to DIO observed in OPA1 BKO mice. Furthermore, like mice lacking OPA1 and GDF15 in BAT [5], DKO mice became hypothermic during cold exposure, suggesting a role for GDF15 signaling through GFRAL to maintain core body temperature in the OPA1 BKO model. In conclusion, our results reveal a new BAT-GDF15-GFRAL axis activated in response to mitochondrial stress that is required to promote resistance to DIO and regulate core body temperature in mice.

## Materials and methods

### Mouse models

**Fig. 1:**
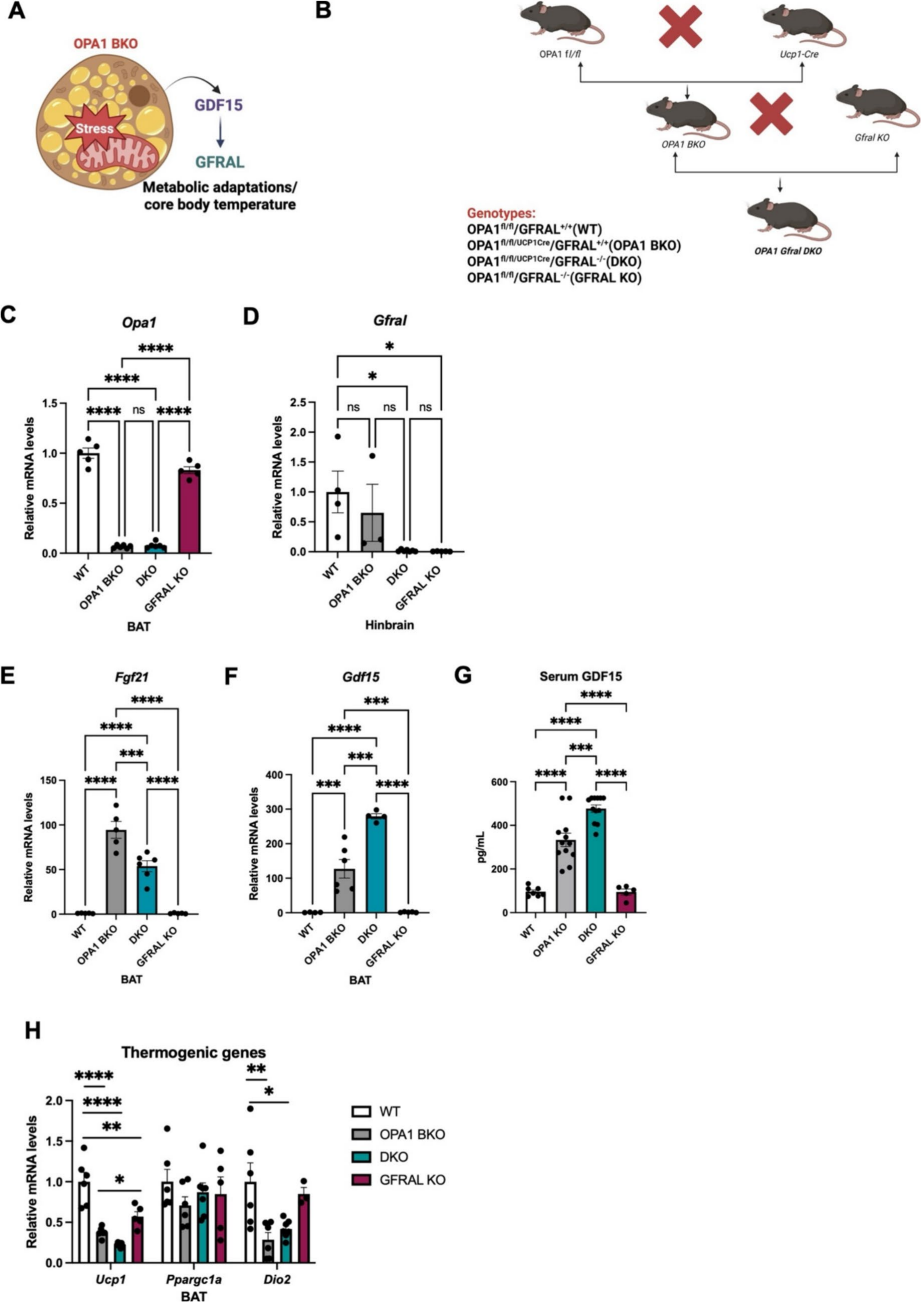
GDF15 induction and thermogenic gene expression. (**A**) Schematic representation of scientific question and (**B**) breeding strategy (created in BioRender. Sood, A., 2025, https://BioRender.com/v0en37f). (**C**) *Opa1* mRNA expression in BAT normalized to *Tbp* (*n* = 4–6). (**D**) *Gfral* mRNA expression in the hindbrain normalized to *Tbp* (*n* = 3–6). (**E**) *Fgf21* mRNA expression in BAT normalized to *Tbp* (*n* = 4–6). (**F**) *Gdf15* mRNA expression in BAT normalized to *Tbp* (*n* = 4–6). (**G**) Circulating GDF15 levels (*n* = 5–12). (H) mRNA levels of thermogenic genes in BAT normalized to *Tbp* (*n* = 3–6). Data presented as mean ± SEM. Significant differences were determined by ordinary one-way ANOVA followed by Tukey’s multiple-comparison test using a significance level of *p* < 0.05. *p < 0.05, **p < 0.01, ***p < 0.001 and ****p < 0.0001

Experiments were performed in male mice on a C57Bl/6 J background. *Opa1*^fl/fl^ were generated as previously described [22]. Transgenic mice expressing Cre recombinase under the control of the *Ucp1* promoter (Tg [Ucp1-cre]1Evdr) [23] were acquired from the Jackson Laboratory (Bar Harbor, ME, #024670). *Gfral*^−/−^ mice were kindly provided by Dr. Randey J. Seeley [24]. Compound mutants were generated by crossing *Opa1*^fl/fl^ mice harboring *Ucp1* Cre with *Gfral*^−/−^ mice for the generation of OPA1 GFRAL double knockout mice (DKO). Wild type (WT) control mice were *Gfral*^+*/*+^ mice homozygous for the *Opa1* floxed alleles but lacking Cre expression. Additional littermate control groups generated by this cross were *Gfral*^+*/*+^ mice homozygous for the *Opa1* floxed alleles and expressing Cre recombinase (OPA1 BKO) and *Gfral*^*−/−*^ mice homozygous for the *Opa1* floxed alleles but lacking the Cre recombinanse (GFRAL KO) (Fig. 1B). Mice were weaned at 3 weeks of age and maintained on a standard chow diet (Harlan Teklad, Indianapolis, IN, #2920X). For DIO experiments, 6-week-old mice were fed a high-fat diet (HFD; 60% kcal from fat; Research Diets, New Brunswick, NJ, #D12492) for 12 weeks. Unless specified, animals were housed at 22˚C under a 12-h light/dark cycle, with ad libitum access to water and either standard chow or HFD. All mouse experiments described in this study were conducted in accordance with NIH animal research guidelines and were approved by the University of Iowa IACUC protocol (#3032294).

### GTTs, ITTs, nuclear magnetic resonance, and serum analysis

Glucose tolerance tests (GTT) were conducted following a 4-h fast for baseline assessments and a 6-h fast for DIO studies after 10 weeks of HFD feeding. Mice received an intraperitoneal injection of glucose (1 g/kg of body weight) [25]. Insulin tolerance tests (ITT) were conducted after a 2-h fast by injecting insulin intraperitoneally (0.75 U/kg body weight; Humulin, Eli Lilly, Indianapolis, IN) after 12 weeks of HFD feeding. Blood glucose was measured using a glucometer at regular time intervals (Glucometer Elite; Bayer, Tarrytown, NY). Insulin and glucose solutions were prepared using sterile 0.9% saline. Plasma insulin levels were measured under ad libitum-fed conditions using a commercially available kit, following the manufacturer’s instructions (Ultra-Sensitive Mouse Insulin ELISA Kit, Chrystal Chem, Downers Grove, IL). Serum GDF15 was measured under ad libitum-fed conditions using commercially available kits according to the manufacturers’ directions (R&D Systems, Minneapolis, MN). Whole-body composition was measured by nuclear magnetic resonance in a Bruker Minispec NF‐50 instrument (Bruker, Billerica, MA).

### Analysis of triglyceride levels

Hepatic triglyceride levels were measured in mice after 12 weeks on HFD using the EnzyChrom Triglyceride Assay Kit (BioAssay Systems, Hayward, CA), as previously described [4, 26].

### Cold exposure

For acute cold exposure experiments, 12-week-old mice were individually housed in rodent environmental chambers (Power Scientific, Inc., Pipersville, PA) set at 30˚C for 7 days. At 8 a.m. on day 8, baseline body temperature (t₀) was measured using a rectal probe (Fisher Scientific, Lenexa, KS). Following this measurement, the chamber temperature was lowered to 4˚C. Once the target temperature was achieved, rectal temperatures were recorded hourly for up to 5 h of cold exposure. After the 5-h cold exposure, animals were euthanized and tissues were collected for molecular analysis.

### RNA extraction and quantitative RT-PCR

Total RNA was isolated from BAT, hindbrain, and iWAT tissues using TRIzol reagent (Invitrogen, Waltham, MA) and further purified with the RNeasy kit (QIAGEN Inc., Germantown, MD). Quantitative RT-PCR was carried out as previously described [4]. Gene expression data were normalized to *Tbp* levels, and results are presented as relative mRNA expression. qPCR primers were either designed using Primer-BLAST or obtained from previously published sources [27]. Primer sequences are provided in Table 1.

**Table 1:** Primer sequences

Gene name	Forward	Reverse
*Fgf21*	TGACGACCAAGACACTGAAGC	TTTGAGCTCCAGGAGACTTTCTG
*Gfral*	TGGAAGACACCTGCCTTACTCC	AGTGGAGGTCATCCATACAAAGAC
*Ucp1*	GTGAAGGTCAGAATGCAAGC	AGGGCCCCCTTCATGAGGTC
*Tbp*	TCTGGAATTGTACCGCAGCTT	CTGCAGCAAATCGCTTGGGA
*Dio2*	AATTATGCCTCGGAGAAGACCG	GGCAGTTGCCTAGTGAAAGGT
*Ppargc1α*	GTAAATCTGCGGGATGATGG	AGCAGGGTCAAAATCGTCTG
*Gdf15*	GAGAGGACTCGAACTCAGAAC	GACCCCAATCTCACCTCTG
*Cpt1β*	TGCCTTTACATCGTCTCCAA	AGACCCCGTAGCCATCATC
*Elovl6*	TCAGCAAAGCACCCGAAC	AGCGACCATGTCTTTGTAGGAG
*Opa1*	ATACTGGGATCTGCTGTTGG	AAGTCAGGCACAATCCACTT

### Western blot analysis

Immunoblotting was conducted as previously described [28]. Roughly 30 mg of frozen BAT or inguinal white adipose tissue (iWAT) were homogenized in 150 μl of lysis buffer, following established methods [4]. HALT protease and phosphatase inhibitors (Thermo Fisher Scientific, Waltham, MA) were added to the lysis buffer just before use. Samples were homogenized using the TissueLyser II (Qiagen Inc., Germantown, MD). Protein lysates were separated by SDS-PAGE and transferred onto nitrocellulose membranes (Millipore Corp., Billerica, MA). Membranes were then incubated with primary antibodies overnight, followed by incubation with secondary antibodies for 1 h under room temperature conditions. UCP1 (1:1000, Abcam, Waltham, MA, Ab10983), tyrosine hydroxylase (1:1000, Cell Signaling Technology, Danvers, MA, #2792) and beta actin (1:1000, Cell Signaling Technology, Danvers, MA, #4967S) were used as primary antibodies and Alexa Fluor anti-rabbit 680 (1:10,000, Invitrogen, #A27042) was used as secondary anitbody. An Odyssey imager (LICORbio, Lincoln, NE) was used to quantify fluorescence. Uncropped immunoblot images are provided in the supplementary file.

### Data analysis

All data are reported as mean ± SEM. To establish statistical differences, ordinary one-way ANOVA along with Tukey’s multiple-comparison test was employed to compare differences between the four groups. A p ≤ 0.05 probability value was considered significantly different. GraphPad Prism Software was used to perform statistical calculations and generate graphs. CalR was used to analyze calorimetry data and for the ANCOVA analysis for the relationship between energy expenditure and body mass [29].

## Results

### GDF15 induction and thermogenic gene expression

To test the effects of GDF15 signaling through GFRAL to mediate the metabolic adaptations and improved thermoregulation observed in OPA1 BKO mice (Fig. 1A), we crossed OPA1 BKO mice with *Gfral*-/- mice (Fig. 1B). To validate appropriate deletion of *Opa1* in BAT and *Gfral* in the hindbrain*,* we performed RT-qPCR in BAT and in the hindbrain of WT, OPA1 BKO, DKO and GFRAL KO mice. *Opa1* mRNA levels were markedly reduced in BAT of both OPA1 BKO and DKO mice relative to WT and GFRAL KO mice (Fig. 1C). *Gfral* levels were significantly reduced in the hinbraind of DKO and GFRAL KO mice relative to WT mice, and trended lower in OPA1 BKO mice, although not statistically significant (Fig. 1D). Following *Opa1* deletion in BAT, we observed increased mRNA expression of *Fgf21* (Fig. 1E) and *Gdf15* (Fig. 1F) in OPA1 BKO and DKO mice relative to WT littermate control mice, but not in GFRAL KO mice [4, 5]. GDF15 serum levels were also significantly elevated in OPA1 BKO and DKO mice relative to WT mice, but not in GFRAL KO mice (Fig. 1G), indicating signaling through GFRAL is not required to mediate induction of GDF15 secretion in OPA1 BKO mice. We also observed impaired thermogenic gene expression in BAT of OPA1 BKO, as previously reported [5], which was maintained in DKO mice (Fig. 1H). These results validate successful deletion of OPA1 and GFRAL in the DKO model and demonstrate that activation of GDF15 as batokine and impaired thermogenic gene expression in BAT of OPA1 BKO mice occur independently of signaling through GFRAL.

### Signaling through GFRAL is dispensable to promote compensatory browning in OPA1 BKO mice under isocaloric conditions

**Fig. 2:**
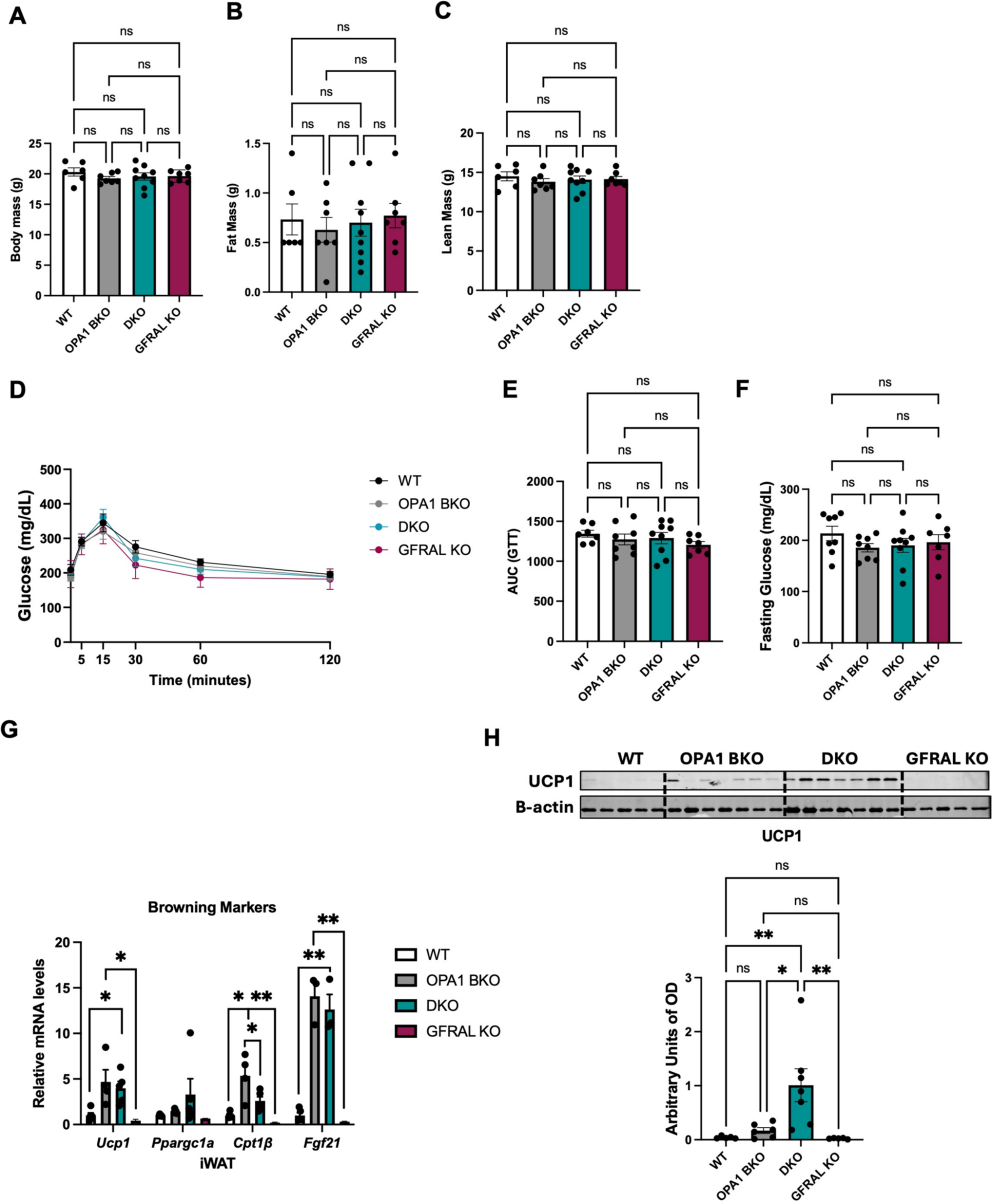
Signaling through GFRAL is dispensable to promote compensatory browning in OPA1 BKO mice under isocaloric conditions. (**A**) Body mass, (**B**) total fat mass, and (**C**) total lean mass in 6-week-old mice (*n* = 6–9). (**D**) Gluocose tolerance test (GTT), (**E**) area under the curve (AUC) for the GTT and (**F**) fasting glucose levels (*n* = 7–9). (**G**) mRNA expression of browning markers in inguinal white adipose tissue (iWAT) (*n* = 3–5). (**H**) Representative immunoblot of UCP1 protein levels in iWAT normalized to β-actin levels and its respective densitometric quantification (*n* = 5–7). OD: optical density. Data presented as mean ± SEM. Significant differences determined by ordinary one-way ANOVA followed by the Tukey’s multiple-comparison test using a significance level of *p* < 0.05. *p < 0.05 and **p < 0.01

To determine changes in baseline metabolic phenotype, we performed NMR and glucose tolerance tests in male mice at 6 weeks of age (prior to starting HFD feeding). Body weight (Fig. 2A), total fat mass (Fig. 2B) and total lean mass (Fig. 2C) were unchanged across genotypes. Additionally, glucose tolerance tests (Figs. 2D, E) and fasting glucose levels (Fig. 2F) were similar among all groups.

Previously, we reported that, following deletion of *Opa1* in BAT, iWAT undergoes compensatory browning, which is dependent on FGF21, but not GDF15 secretion from BAT under baseline conditions [4, 5]. Indeed, DKO mice had increased compensatory browning of iWAT, as shown by induced mRNA expression of thermogenic markers (Fig. 2G) and higher UCP1 protein levels in iWAT (Fig. 2H). Importantly, GFRAL KO had similar thermogenic gene expression and UCP1 levels as WT mice (Fig. 2G and H). This phenotype recapitulates our findings in mice simultaneously lacking *Opa1* and *Gdf15* in BAT, which lacked the induction of GDF15 circulating levels observed in OPA1 BKO mice, but had preserved iWAT browning [5]. Together, these findings indicate that GDF15 signaling through GFRAL is largely dispensable to promote metabolic changes in OPA1 BKO under baseline conditions, corroborating our previous findings [5].

### Signaling through GFRAL partially mediates resistance to DIO in OPA1 BKO mice

**Fig. 3:**
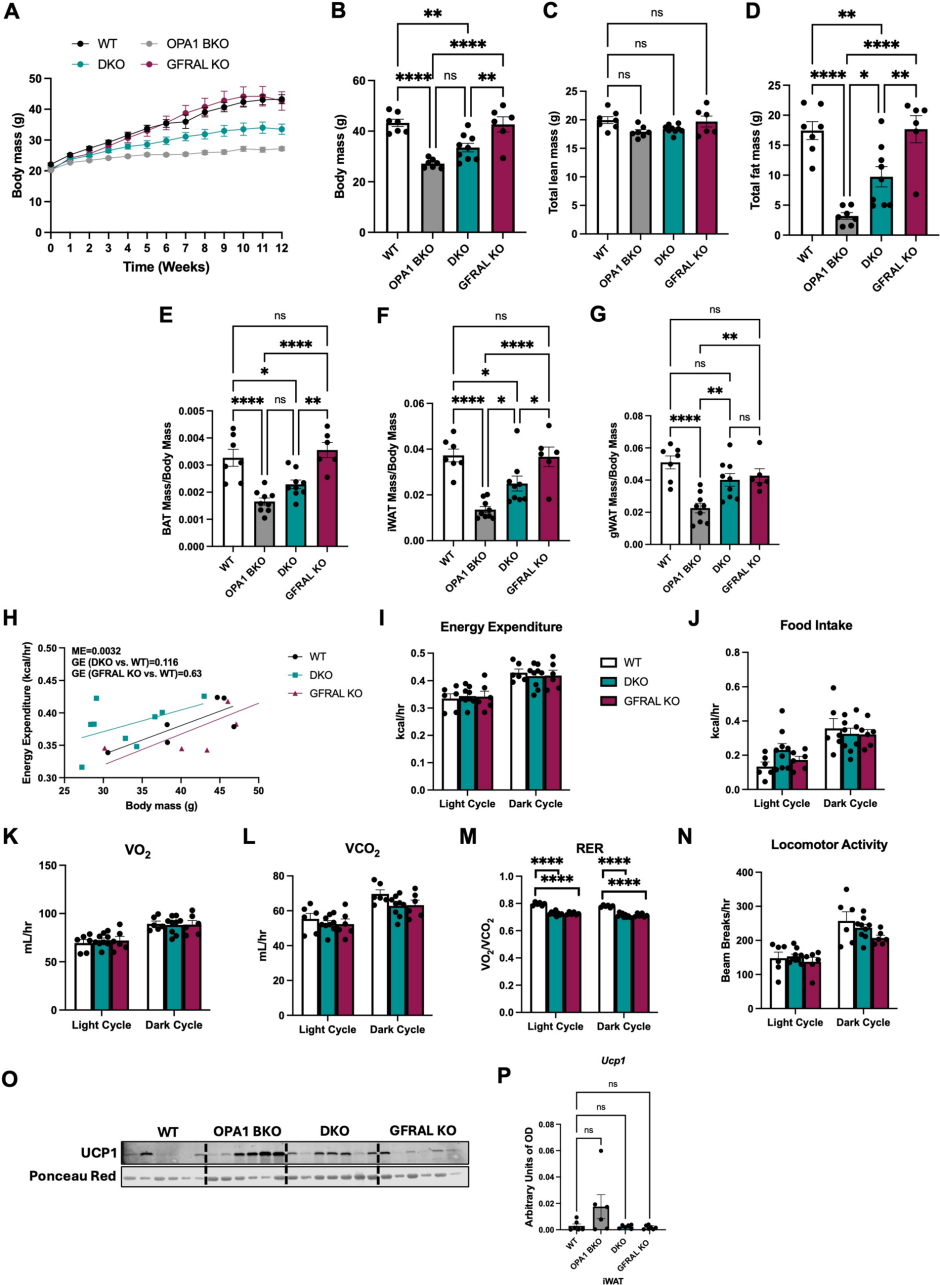
Signaling through GFRAL partially mediates resistance to DIO in OPA1 BKO mice. (**A**) Body weight over time, (**B**) final body mass, (**C**) total lean mass, and (**D**) total fat mass (*n* = 6–9). (**E**) BAT mass normalized to body mass (*n* = 6–9). (**F**) iWAT mass normalized to body mass (*n* = 6–9). (**G**) gWAT mass normalized to body mass (*n* = 6–9). (**H-N**) Indirect calorimetry. (**H**) Regression plot of energy expenditure as a function of body mass (*n* = 6–9). (**I-N**) Data represented as the average for the light and dark cycles during the last 48 h of data recording. (**I**) Energy Expenditure (*n* = 6–9). (**J**) Food Intake (*n* = 6–9). (**K**) Oxygen consumption (VO_2_) (*n* = 6–9). (**L**) Carbon dioxide production (VCO_2_) (*n* = 6–9). (**M**) Respiratory exchange ratio (*n* = 6–9). (**N**) Locomotor Activity (*n* = 6–9). (**O**) Representative immunoblot of UCP1 protein levels in iWAT normalized to ponceau red levels and (**P**) its respective densitometric quantification (*n* = 6–7). OD: optical density. Data presented as mean ± SEM. Significant differences determined by ordinary one-way ANOVA followed by the Tukey’s multiple-comparison test using a significance level of *p* < 0.05. ANCOVA analysis was used to determine the mass effect (ME) and group effect (GE) in the regression plot. *p < 0.05, **p < 0.01, ***p < 0.001 and ****p < 0.0001

To test whether signaling through GFRAL is required to mediate GDF15’s effect on weight gain resistance, WT, OPA1 BKO, DKO and GFRAL KO mice were subjected to HFD for 12 weeks. Over time, OPA1 BKO stayed resistant to weight gain relative to all groups, recapitulating our previous findings [4, 5], whereas GFRAL KO mice gained weight at a similar rate as WT mice (Figs. 3A, B). Interestingly, DKO mice had an intermediate phenotype, having significantly lower final body weight relative to WT mice (Figs. 3A, B), while still accumulating more fat mass than OPA1 BKO mice (Figs. 3D, F and G). Total lean mass was unchanged across genotypes (Fig. 3C), while fat mass was significantly reduced in both OPA1 BKO and DKO mice relative to WT, but remained elevated in DKO mice when compared to OPA1 BKO (Fig. 3D). When normalized to body weight, BAT (Fig. 3E), iWAT (Fig. 3F) and gWAT (Fig. 3G) weights were significantly lower in OPA1 BKO and DKO mice as compared to WT littermates, but iWAT (Fig. 3F) and gWAT (Fig. 3G) weights were significantly higher in DKO mice versus OPA1 BKO mice, in agreement with total fat mass data (Fig. 3D). In our previous study, we showed by ANCOVA analysis that OPA1 BKO mice had higher energy expenditure as a function of body mass, compensatory hyperphagia and increased respiratory exchange ratio (RER) relative to their WT littermate controls. These changes were abrogated when GDF15 was deleted in the OPA1 BKO background [5]. To determine if these effects were dependent on GFRAL, we performed indirect calorimetry in WT, DKO and GFRAL KO mice. Our data showed no changes in energy expenditure (Fig. 3H, I), food intake (Fig. 3J), oxygen consumption rates (Fig. 3K) or carbon dioxide production (Fig. 3L) across genotypes. RER was significantly lower in both DKO and GFRAL KO mice compared to WT controls (Fig. 3L). Finally, locomotor activity (Fig. 3N) was largely unchanged across genotypes. In OPA1 BKO mice, higher energy expenditure correlated with increased browning of iWAT under HFD conditions, an effect mediated by GDF15 [5]. Although not statistically significant, here we show that DKO mice tend to have attenuated iWAT browning under obesogenic conditions relative to OPA1 BKO mice (Fig. 3O, P), suggesting that GDF15 signaling through GFRAL is required to maintain iWAT browning in OPA1 BKO mice fed HFD.

### GFRAL deletion attenuates the improvements in glucose homeostasis and insulin sensitivity in OPA1 BKO mice fed HFD

**Fig. 4:**
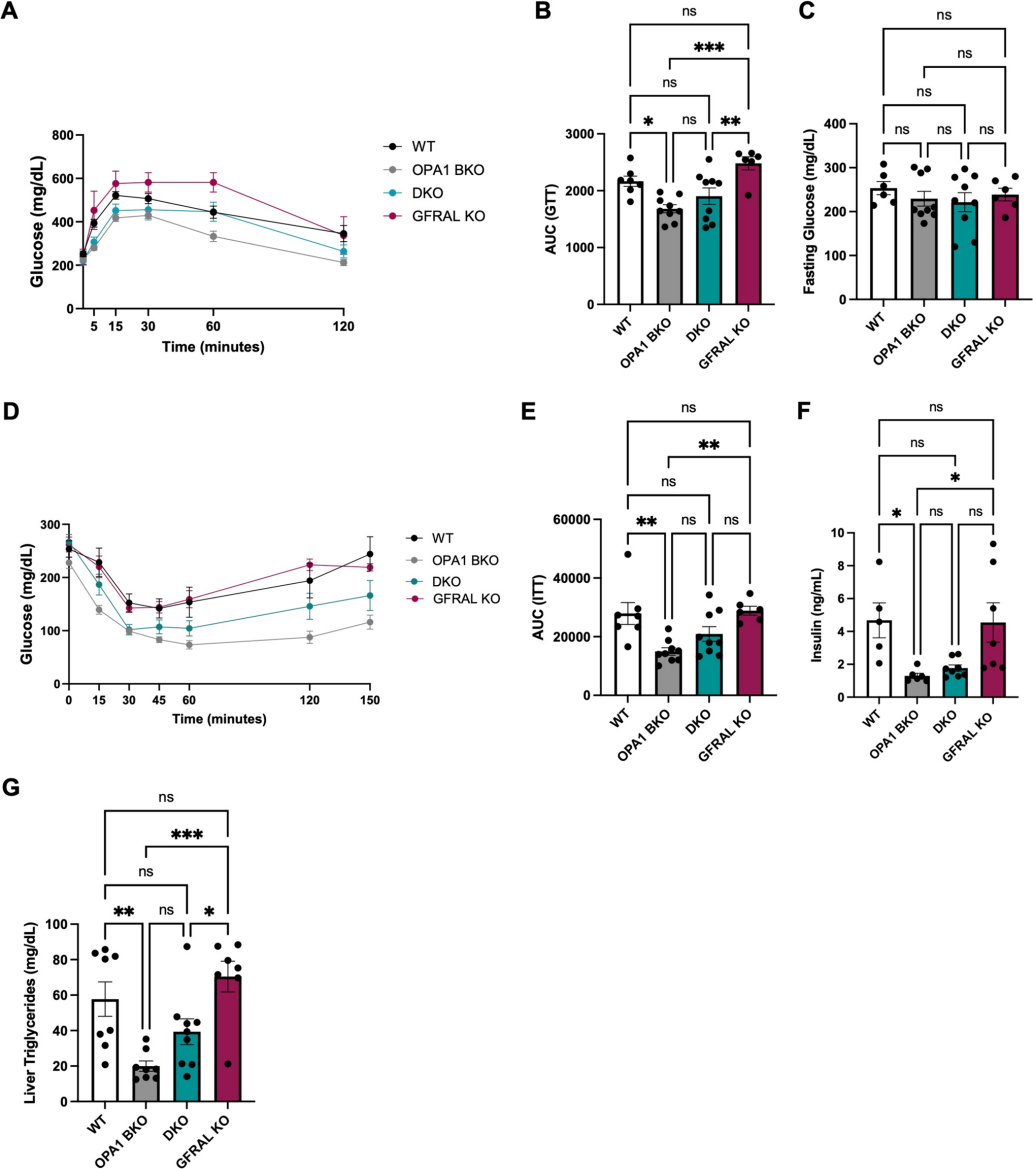
GFRAL deletion attenuates the improvements in glucose homeostasis and insulin sensitivity in OPA1 BKO mice fed HFD. (**A**) Glucose tolerance test (GTT) (*n* = 6–9). (**B**) Area under the curve (AUC) for the GTT (*n* = 6–9). (**C**) Fasting glucose levels (*n* = 6–9). (**D**) Insulin tolerance test (ITT) (*n* = 7–9). (**E**) AUC for the ITT (*n* = 7–9). (**F**) Serum insulin levels (*n* = 5–8). (**G**) Liver triglyceride levels (*n* = 7–9). Data presented as mean ± SEM. Significant differences determined by ordinary one-way ANOVA followed by Tukey’s multiple-comparison test using a significance level of *p* < 0.05. *p < 0.05, **p < 0.01 and ***p < 0.001

To determine changes in systemic glucose homeostasis and insulin sensitivity, GTTs and ITTs were conducted in WT, OPA1 BKO, DKO and GFRAL KO groups following HFD feeding. We previously showed that OPA1 BKO mice had improved glucose tolerance and insulin sensitivity relative to WT mice on HFD [4]. Here, we recapituale these findings by showing reduced area under the curve (AUC) for the GTT and ITT, and reduced insulin levels in OPA1 BKO mice relative to the WT controls (Fig. 4A-F). Despite having reduced fat mass relative to WT control mice, DKO mice lacked the improvement in glucose homeostasis, as shown by unchanged glucose tolerance test (Fig. 4A, B) and fasting glucose levels compared to WT control mice (Fig. 4C). ITTs (Fig. 4D, E) and insulin levels (Fig. 4F) were also similar between WT and DKO mice. Finally, we previously showed that OPA1 BKO mice had attenuated diet-induced hepatic steatosis [4], which was prevented when GDF15 was deleted in BAT [5]. Here, we show that this phenotype requires signaling through GFRAL, as DKO mice have similar liver triglyceride accumulation as WT mice, while OPA1 BKO mice had lower levels of liver triglycerides (Fig. 4G). Finally, GFRAL KO mice had similar glucose homeostasis, insulin sensitivity and hepatic triglyceride levels compared to WT mice.

### Signaling through GFRAL is required to maintain core body temperature in cold exposed OPA1 BKO mice

**Fig. 5:**
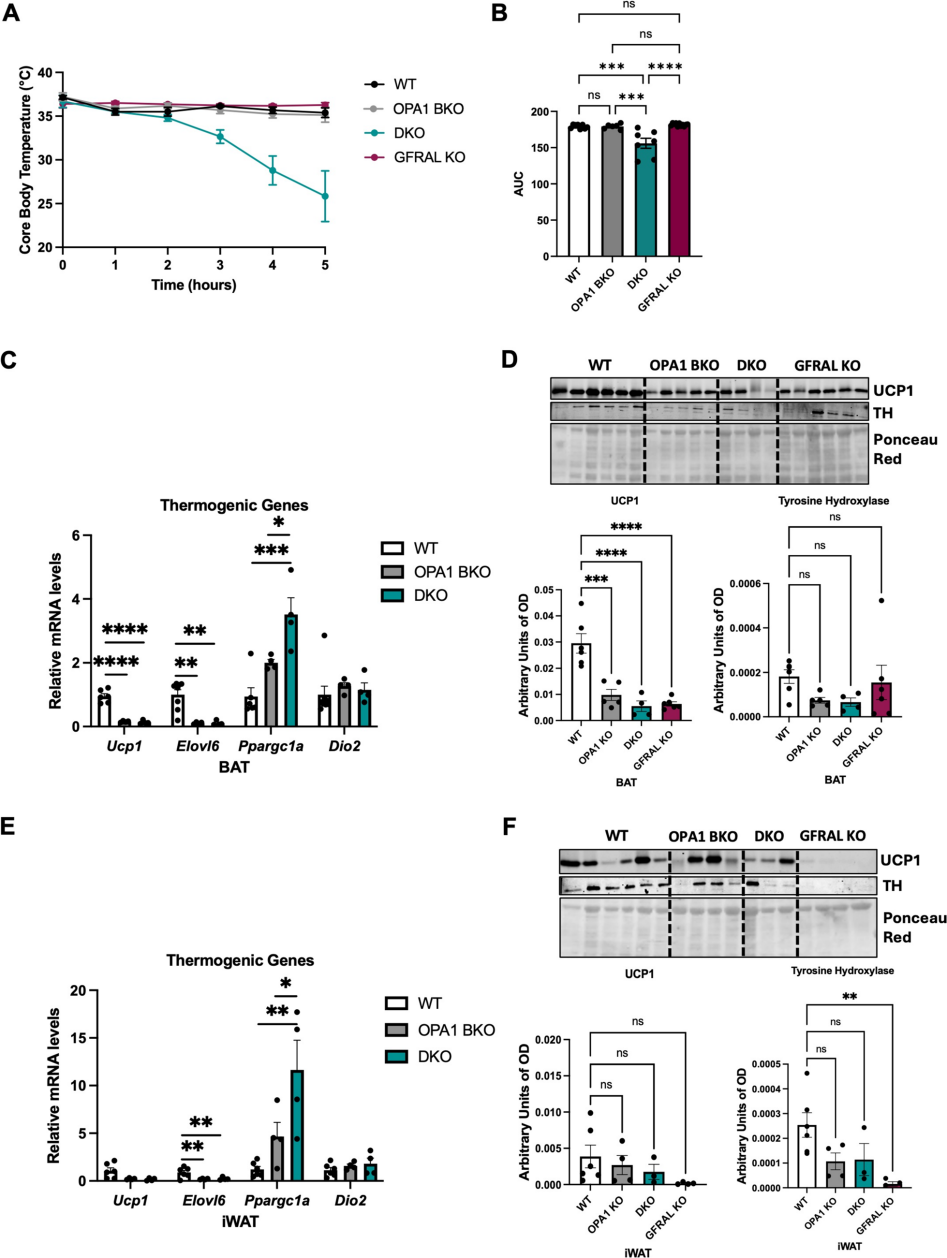
Signaling through GFRAL is required to maintain core body temperature in cold exposed OPA1 BKO mice. (**A**) Core body temperature collected hourly with a rectal probe (*n* = 6–11). (**B**) Area under the curve (AUC) for core body temperature (*n* = 6–11). (**C**) mRNA expression of thermogenic genes in BAT normalized to *Tbp* (*n* = 4–8). (**D**) Representative immunoblot of UCP1 and tyrosine hydroxylase (TH) protein levels in BAT normalized to ponceau red levels and their respective densitometric quantifications (*n* = 4–6). (**E**) mRNA expression of thermogenic genes in iWAT normalized to *Tbp* (*n* = 4–6). (**F**) Representative immunoblot of UCP1 and TH protein levels in iWAT normalized to ponceau red levels and their respective densitometric quantifications (*n* = 3–6). OD: optical density. Data presented as mean ± SEM. Significant differences determined by ordinary one-way ANOVA followed by Tukey’s multiple-comparison test, using a significance level of *p* < 0.05. *p < 0.05, **p < 0.01, ***p < 0.001 and ****p < 0.0001

We have previously shown that OPA1 BKO mice had improved thermoregulation in response to cold relative to WT control mice [4]. Remarkably, GDF15 deletion in the OPA1 BKO background rendered mice cold intolerant [5]. Here, we show that GDF15 signaling through GFRAL is required to maintain core body temperature in OPA1 BKO mice, with GFRAL deletion resulting in cold-induced hypothermia (Fig. 5A, B). Interestingly, GFRAL KO mice had similar core body temperature as WT mice, indicating GFRAL is dispensible for body temperature regulation during an acute cold stress. To determine if changes in thermogenic gene program contributed to this phenotype, we performed mRNA analysis of a subset of thermogenic markers in BAT following cold exposure. We observed reduced expression of *Ucp1* and *Elovl6* (Fig. 5C), while *Ppargc1a* levels were similarly increased in both OPA1 BKO and DKO relative to WT mice following cold exposure. Likewise, UCP1 protein levels in BAT were equivalently reduced in all genotypes relative to WT mice (Fig. 5D). To estimate sympathetic activation, we measured tyrosine hydroxylase (TH) levels, but found no statistical differences across groups (Fig. 5D). We next evaluated if impaired cold-induced iWAT browning might underly the hypothermia phenotype observed in DKO mice. Surprisingly, changes in thermogenic markers in iWAT were similar between OPA1 BKO and DKO mice (Fig. 5E). UCP1 protein levels in iWAT were variable and not statistically different across groups (Fig. 5F). Noteworthy, TH levels were significantly reduced in iWAT of GFRAL KO mice relative to WT controls, but was unchanged in OPA1 BKO and in DKO mice (Fig. 5F).

## Discussion

The role of GDF15 signaling in obesity has been well established in rodents and non-human primates [10, 13–16, 30]. GDF15’s receptor and coreceptor, GFRAL and RET, respectively, were characterized in 2017 through a series of studies from different groups and were shown to localize to strict areas of the hindbrain [13–16]. GDF15 binding to GFRAL regulates energy balance primarily by reducing food intake, although a recent study showed that GDF15 signaling through GFRAL can also regulate changes in energy expenditure by increasing futile calcium cycling in skeletal muscle in mice [30]. Although GFRAL has been the only receptor for GDF15 thoroughly validated to date, it has been proposed that GDF15 may mediate its effects through additional targets, especially in peripheral tissues, in a GFRAL-independent manner [17, 18, 31–35]. Therefore, better characterization of GFRAL-dependent and -independent effects of GDF15 is critical to inform GDF15-based therapies. Our previous studies in mice lacking OPA1 in brown adipocytes (OPA1 BKO) revealed that mitochondrial stress induces GDF15 secretion as a batokine, which is required to attenuate weight gain in a model of DIO, primarily by regulating changes in energy expenditure but not food intake. Furthermore, GDF15 promoted maintainance of core body temperature in cold-exposed OPA1 BKO mice [4, 5]. Here we tested the hypothesis that signaling through GFRAL is required to convey these GDF15-mediated effects in OPA1 BKO mice.

We first assessed the baseline metabolic phenotype of DKO mice relative to WT controls, OPA1 BKO and GFRAL KO mice. GFRAL deletion in the OPA1 BKO mice did not affect baseline metabolic parameters such as body weight, body composition and glucose homeostasis. Moreover, our data show that GFRAL is dispensable to drive compensatory browning of WAT in young OPA1 BKO mice fed regular chow, a phenotype that was also observed in mice lacking OPA1 and GDF15 in BAT (OPA1/GDF15 DKO) [5], and was previously attributed to BAT-derived FGF21 [4]. Our earlier study also showed that OPA1 BKO mice are remarkably resistant to DIO [4]. Here, we recapitulated these findings and uncovered that GFRAL contributes to prevent fat mass accumulation in OPA1 BKO mice under obesogenic conditions. This phenotype is similar to that observed in mice lacking OPA1 and GDF15 in BAT (OPA1/GDF15 DKO), which only partially lost resistance to DIO [5]. Importantly, in our previous studies, we concluded that resistance to DIO in OPA1 BKO mice was caused by increases in energy expenditure, which were abrogated in the absence of GDF15 [5]. Similarly, DKO mice have similar energy expenditure relative to WT control mice. In OPA1 BKO mice, increased energy expenditure correlated with induced WAT browning, an effect that was attenuated in both OPA1/GDF15 [5] and DKO mice fed HFD. These data suggest a role for GDF15-GFRAL siganling in mediating WAT browning in OPA1 BKO mice under obesogenic conditions. This is in agreement with a study showing that, following treatment with GDF15, DIO mice tend to have increased energy expenditure, which is accompanied by increased UCP1 expression in WAT, suggesting GDF15-induced browning [36]. Based on our previous work [4–6] and the new indirect calorimetry data presented here, we conclude that obesity was attenuated in DKO mice due to lack of GDF15-GFRAL-mediated increases in energy expenditure rather than changes in food intake. Our current findings reinforce the notion that GFRAL effects on energy balance go beyond regulation of food intake and include modulation of energy expenditure in the context of DIO and mitochondrial stress, potentially via increased browning of WAT.

Interestingly, although DKO had reduced fat mass accrual relative to WT mice following HFD-feeding, glucose homeostasis, systemic insulin sensitivity and hepatic steatosis were not improved in these mice, contrasting with OPA1 BKO. Early studies showed that GDF15 overexpression and treatment with recombinant GDF15 leads to improvements in glucose tolerance and insulin sensitivity in DIO models, which are associated with reductions in body weight [37, 38]. In contrast, GDF15 global kockout mice have a slight increase in body weight, which is associated with impaired glucose homeostasis and increased hepatic triglyceride levels during DIO [39]. More recently, a study showed that GDF15 pharmacological administration can regulate insulin signaling and glucose utilization independent of weight loss [40]. Our data suggest that signaling through GFRAL might have direct effects on glucose homeostasis, insulin tolerance and liver lipid metabolism in OPA1 BKO mice. However, we cannot discard the possibility that the higher fat mass accumulation in DKO is sufficient to prevent the improvements in these metabolic parameters observed in OPA1 BKO mice. Noteworthy, under the conditions investigated in our study, global GFRAL deletion alone did not cause changes in body weight, food intake, glucose homeostasis or hepatic steatosis when compared to WT control mice. These data contrast with previous studies in GFRAL global KO mice [13, 16], and may reflect differences in mouse genetic background and experimental design, including the diet selection and duration of HFD feeding.

Although a link between GDF15 treatment and browning of WAT has been described in DIO mice [36], whether GDF15 signaling through GFRAL is required to modulate cold-induced thermogenesis is less clear. A recent study showed that overexpression of GDF15 in male mice leads to higher rectal temperature and thermogenic gene activation in BAT and iWAT upon cold exposure [41]. In our previous study, we showed that GDF15 deletion in the OPA1 BKO background rendered mice cold intolerant, suggesting a role for GDF15 on thermoregulation in this model [5]. OPA1/GFRAL DKO mice also became severely hypothermic when subjected to acute cold stress, confirming that signaling through GFRAL is required to maintain core body temperature in cold-exposed OPA1 BKO mice. Noteworthy, expression of thermogenic markers, and UCP1 protein levels in BAT and iWAT were similarly altered in cold-exposed OPA1 BKO and DKO mice, suggesting UCP1-independent mechanisms might be affected by GFRAL deletion in OPA1 BKO mice, contributing to cold-induced hypothermia. Indeed, signaling through GFRAL has been shown to induce futile calcium cycling in the skeletal muscle of DIO mice treated with recombinant GDF15 [30]. Whether this mechanism is also induced following acute cold in a GFRAL-dependent manner and the role of UCP1-independent thermogenesis in OPA1 BKO mice will require further investigation. Moreover, additional contributors to thermoregulation that could arise from developmental remodeling, including adrenergic BAT output or vasomotor responses were not investigated in the present study. Therefore, our conclusions in mice with global, lifelong GFRAL deletion are limited, as developmental changes in thermoregulatory circuits cannot be excluded. Nonetheless, the current data combined with our pervious study [5] provide compelling evidence that GDF15-GFRAL siganling plays an essential role in regulating adaptations to cold exposure in mice undergoing mitochondrial stress via yet to be identified mechanisms. Finally, although GFRAL KO mice are able to maintain core body temperature in response to acute cold stress, UCP1 levels in BAT and tyrosine hyodroxylase levels in iWAT were reduced in these mice, suggesting impairments in sympathetic activation and BAT thermogenesis.

## Conclusions

Our work demonstrates that GDF15 signaling through GFRAL partially mediates resistance to DIO and is critical to maintain core body temeperature during cold exposure in OPA1 BKO mice. Noteworthy, DKO mice have attenuated WAT browning during obesogenic conditions, likely contributing to increased fat mass accrual. Importantly, these effects observed in the context of OPA1 deficiency were absent in GFRAL KO mice, demonstrating different adaptations exist in response to mitochondrial versus physiological stress. In conclusion, our study reveals a new BAT-GDF15-GFRAL axis that regulates systemic metabolic adapaptions in the context of mitochondrial stress.

## Supplementary Information

Below is the link to the electronic supplementary material.

Supplementary file1 (JPG 606 KB)

## Data Availability

All datasets generated and/or analyzed during the current study are presented in the article, or are available from the corresponding author upon reasonable request.
