# Supplementary figures and images for: GFRAL is required to mediate changes in systemic metabolism in response to mitochondrial stress in brown adipose tissue
Source: J Mol Med (Berl). 2026 Apr 14;104(1):64. doi: 10.1007/s00109-026-02671-z (PMC13076437; doi:10.1007/s00109-026-02671-z)

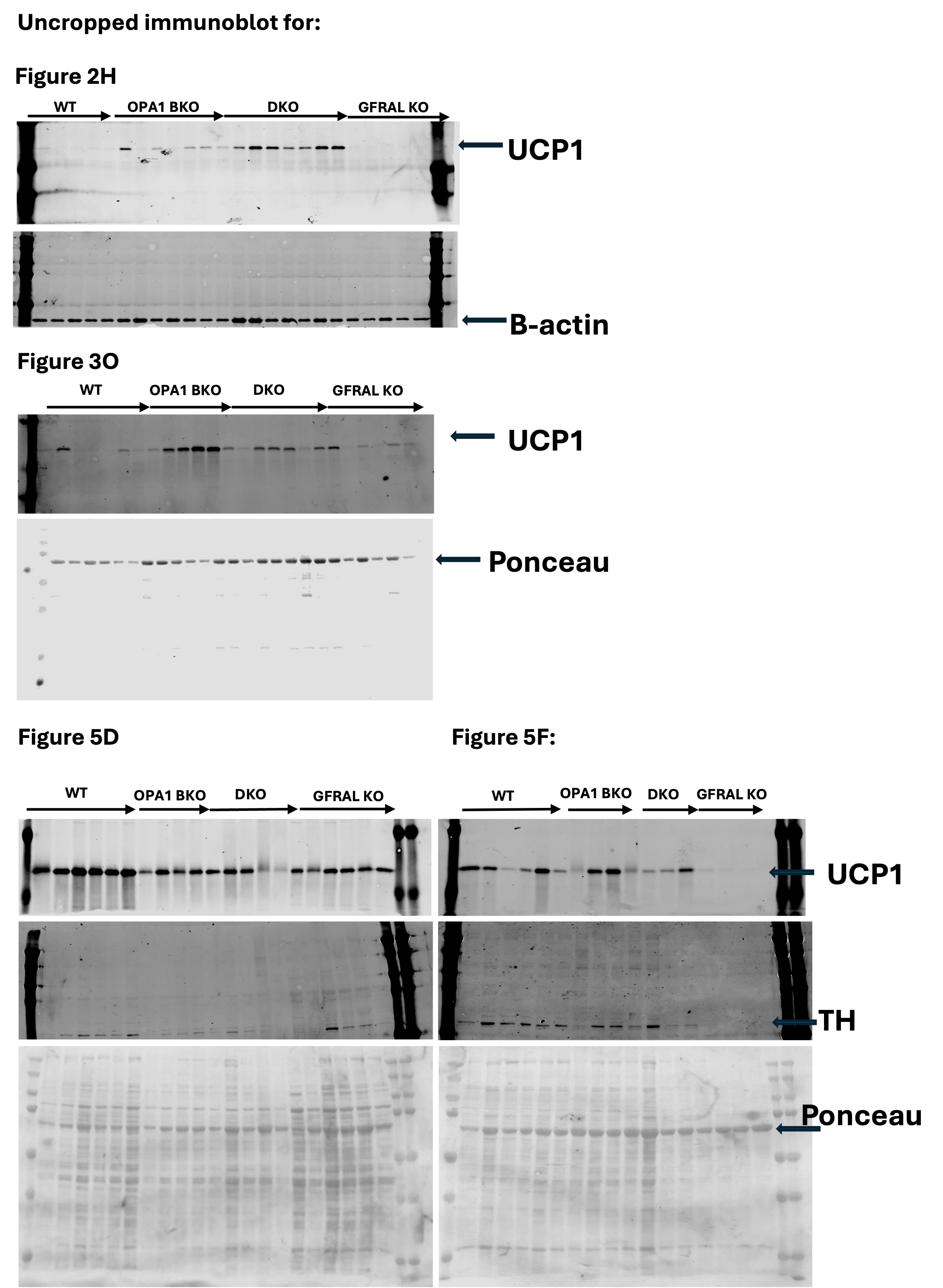

Supplement: Supplementary file 1 — Supplementary file1 (JPG 606 KB) [file 109_2026_2671_MOESM1_ESM.jpg]
